# Approximating Human-Level 3D Visual Inferences With Deep Neural Networks

**DOI:** 10.1162/opmi_a_00189

**Published:** 2025-02-16

**Authors:** Thomas P. O’Connell, Tyler Bonnen, Yoni Friedman, Ayush Tewari, Vincent Sitzmann, Joshua B. Tenenbaum, Nancy Kanwisher

**Affiliations:** Brain & Cognitive Sciences, MIT, Cambridge, MA, USA; EECS, University of California, Berkeley, Berkeley, CA, USA; CSAIL, MIT, Cambridge, MA, USA; Brain & Cognitive Sciences, CSAIL, MIT, Cambridge, MA, USA

**Keywords:** 3D shape perception, deep neural networks, neural fields, psychophysics

## Abstract

Humans make rich inferences about the geometry of the visual world. While deep neural networks (DNNs) achieve human-level performance on some psychophysical tasks (e.g., rapid classification of object or scene categories), they often fail in tasks requiring inferences about the underlying shape of objects or scenes. Here, we ask whether and how this gap in 3D shape representation between DNNs and humans can be closed. First, we define the problem space: after generating a stimulus set to evaluate 3D shape inferences using a match-to-sample task, we confirm that standard DNNs are unable to reach human performance. Next, we construct a set of candidate 3D-aware DNNs including 3D neural field (Light Field Network), autoencoder, and convolutional architectures. We investigate the role of the learning objective and dataset by training single-view (the model only sees one viewpoint of an object per training trial) and multi-view (the model is trained to associate multiple viewpoints of each object per training trial) versions of each architecture. When the same object categories appear in the model training and match-to-sample test sets, multi-view DNNs approach human-level performance for 3D shape matching, highlighting the importance of a learning objective that enforces a common representation across viewpoints of the same object. Furthermore, the 3D Light Field Network was the model most similar to humans across all tests, suggesting that building in 3D inductive biases increases human-model alignment. Finally, we explore the generalization performance of multi-view DNNs to out-of-distribution object categories not seen during training. Overall, our work shows that multi-view learning objectives for DNNs are necessary but not sufficient to make similar 3D shape inferences as humans and reveals limitations in capturing human-like shape inferences that may be inherent to DNN modeling approaches. We provide a methodology for understanding human 3D shape perception within a deep learning framework and highlight out-of-domain generalization as the next challenge for learning human-like 3D representations with DNNs.

## INTRODUCTION

When we look at an object, we do more than recognize its category or position in space. We perceive the orientation and curvature of its visible surfaces, and from this we can infer its global 3D shape (Ullman, 1987). This ability enables us to imagine what any given object might look like from a different perspective, for example, or to understand an object’s affordances, such that we can grasp or interact with objects, even when they have arbitrary, unfamiliar shapes (Gibson, 2014). Remarkably, humans can infer 3D shape from a single image (Hassanin et al., 2021). Inferring these underlying properties from a single image is an ill-posed problem (Pizlo, 2001)—that is, any given image is consistent with multiple interpretations of it’s underlying geometry. As such, inferring object geometry depends on our ability to resolve formidable computational challenges. A rich body of empirical evidence exists to understand this ability, and many theories have been proposed to account for these data (Bülthoff & Mallot, 1988; Janssen et al., 2000; Kersten et al., 2004; Marr, 2010; Shepard & Metzler, 1971; Ullman, 1979; Wertheimer, 1938). In recent years, there has been a renewed interest in developing formal, computational accounts of 3D shape perception, including analysis-by-synthesis approaches (Yildirim et al., 2020; Yuille & Kersten, 2006). Despite some promising progress, the factors that lead to human-level 3D understanding in computational models remain unclear.

Over the past 10 years, deep neural networks (DNNs) have emerged as a promising methodological framework for studying primate vision (Doerig et al., 2023; Kriegeskorte, 2015; Yamins & DiCarlo, 2016). DNNs are appealing as models of visual processing, in part, because their architecture was loosely inspired by the organization of the primate visual system. Critically, DNNs are ‘stimulus-computable’ models, such that they make predictions of behavioral and neural responses directly from images (Khaligh-Razavi & Kriegeskorte, 2014; Rajalingham et al., 2018; Schrimpf et al., 2020; Yamins et al., 2014) and currently provide the most quantitatively accurate accounts of neural responses and behaviors that depend on the ventral visual stream (Doerig et al., 2023; Kriegeskorte, 2015; Yamins & DiCarlo, 2016). Nonetheless, significant gaps remain between standard DNNs and human performance on many visual tasks, most notably inferring the shape of objects. ImageNet-trained CNNs are biased to classify images based on texture, for example, whereas humans show a strong shape classification bias (Baker et al., 2018; Geirhos et al., 2018; Hermann et al., 2020). In 3D shape tasks that require matching images that depicting the same object from different viewpoints, humans outperform standard CNNs by a wide margin (Bonnen et al., 2021; Rajalingham et al., 2018). It is unclear what components of this modeling approach (e.g., model architecture, training objective, or dataset) leads to the misalignment with human 3D shape inference abilities.

The limitations of standard DNNs trained on large-scale datasets (e.g., ImageNet) in capturing the 3D shape of objects and scenes is well recognized in the computer vision community (Abbas & Deny, 2023; Alcorn et al., 2019; Cooper et al., 2021; Geirhos et al., 2018; Reizenstein et al., 2021). Several computational methods have recently been developed to better infer 3D geometry in DNNs (Xie et al., 2022). Unlike standard DNNs, these models’ architectures and training procedures were designed to directly account for the 3D properties of objects and scenes. One set of recent 3D-aware architectures, 3D neural fields, learn a continuous function that maps xyz or ray coordinates from a 3D volume to shape and/or color, given the object’s pose. 3D neural fields are typically trained with a multi-view rendering objective, in which the model computes the geometry from some set of input images and outputs renders of the object or scene from novel viewpoints not included in the inputs. The most common class of 3D neural fields, NEural Radiance Fields (NERFs), are optimized directly on many views of an individual object or scene, and can be used to create near photo-realistic 3D models (Mildenhall et al., 2021). Other methods, such as conditional 3D neural fields, learn a generalizable shape space which can recover the global 3D shape of objects from a single image (Sitzmann et al., 2021; Yu et al., 2021). The improved performance of these models reconstructing 3D shape from images raises the question of whether they might be more aligned with human 3D inference abilities.

Here, we evaluate what properties of DNNs are necessary for them to make similar 3D shape inferences as humans. First, we construct a 3D match-to-sample task in which human participants match images depicting the same object from two different viewpoints. As expected, we observe a large gap between standard ImageNet-trained DNNs and humans. Next, we train a set of DNNs to investigate the role of architecture and learning objective on model alignment to humans. This set includes 3D Light Field Networks (LFNs), convolutional neural networks (CNNs, e.g., resnet50), and autoencoder models, each trained with both single-view and multi-view learning objectives. We find that DNNs trained with a multi-view objective make similar 3D shape inferences to humans. We create a series of ‘adversarial’ conditions for models, and find that the performance of humans and multi-view DNNs is robust even for these difficult trials. Notably, 3D-LFNs show the highest alignment to humans. Finally, we characterize the generalization of multi-view DNNs to out-of-distribution object categories not included in training. Overall, we demonstrate that DNNs are capable of making similar 3D shape judgements as humans when trained with a multi-view learning objective and tested on objects that overlap with the training distribution. Our approach provides a framework for studying human 3D shape inferences alongside deep learning models, identifying out-of-domain generalization to novel shape categories as a primary challenge for future research.

## RESULTS

### 3D Shape Judgements in Humans and ImageNet DNNs

**Figure 1.**
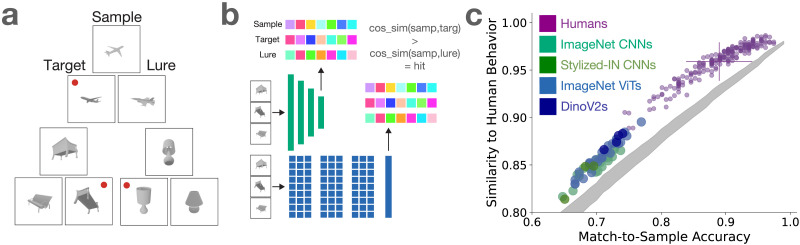
a.) Example trials from the match-to-sample task used to probe 3D shape representations in humans and models. Participants saw all three images concurrently, and their task was choose which of the bottom two objects (target and lure) depict the top object (sample) from a new viewpoint. For visualization purposes in this figure, the correct responses are indicated with a red dot. b.) Model procedure for match-to-sample tasks with baseline ImageNet models. For a given trial, the sample, target, and lure images were separately run through the network, and unit activity from the final layer before the classification layer were extracted as features. The cosine similarity between the sample/target features and sample/lure features was computed, and if the sample/target similarity was higher the trial is counted as correct. The green schematic shows the process for CNNs and the blue for ViTs. c.) Match-to-sample results for within-category 3D shape judgements. Each purple dot is a single human participant (*N* = 200). For human data, the y-axis encodes the average self-similarity of one held out participant to the mean of all other human participants (noise-ceiling). The errorbars along the y-axis show one standard deviation in accuracies across participants, and the errorbars along the y-axis show one standard deviation in the leave-one-subject-out noise-ceiling across participants. The shaded grey distribution is a random observer baseline that shows the expected range of trial-wise similarity to humans across accuracies if the correct and incorrect responses are randomly distributed across trials (see Methods - Random Observer Baseline).

To probe 3D shape representations in humans and DNNs, we use a multi-view match-to-sample task. For the human experiments, three images are shown concurrently in each trial (Figure 1a) and remain on the screen until the participant makes their response. The top image (sample) depicts an object from one viewpoint. For the two images below, one depicts the same object as the sample from a different viewpoint (target) and the other depicts a different object from the same category (lure). Viewpoints for all objects were sampled randomly from a sphere around the object. The task for humans is to simply choose which of the target and lure images is the same object depicted in the sample image. Similar tasks have been used previously to probe for 3D shape representations in both models and humans (Bonnen et al., 2021; Rajalingham et al., 2018). Rajalingham et al. (2018) showed that ImageNet CNNs are aligned to human shape matching judgements when the target and lure objects are drawn from different categories, but show a large gap to humans when the target and lure objects are both from the same category. This provides the motivation for our design where target and lure pairs are always drawn from the same category, so solving the task requires more fine-grained shape judgements rather than being solvable via categorization. For all experiments presented in this paper, we collect human behavioral responses (*N* = 200) online using Prolific (www.prolific.co).

Stimuli were rendered from objects in the ShapeNet dataset (Chang et al., 2015). We use a subset of ShapeNet that has 30565 training objects and 8736 test objects drawn from 13 manmade categories. Match-to-sample trials were generated by randomly pairing two test objects from the same category, then creating two trials with those objects’ renders (one where object A is the sample/target and object B is lure, and one where object B is the sample/target and object A is the lure). Each participant only saw one trial for a given pair so all objects were novel throughout the experiment. Examples can be seen in Figure 1a.

To complete the analogous task on DNNs (Figure 1b), each of the three images for a given trial are fed into a given network and unit activity was extracted from the penultimate layer. The match-to-sample task is completed via a similarity-based measure; cosine similarity is computed between the sample/target and sample/lure unit activity, and the trial is counted as correct if the sample-target similarity is higher than the sample-lure similarity. For the baseline DNN model zoo, we use 25 CNNs from PyTorch (Reizenstein et al., 2021) and 25 ViTs from the timm package (timm.fast.ai), all pretrained for object classification on ImageNet. We also test 3 resnet50 CNNs trained on Stylized-ImageNet (Stylized-IN), a version of ImageNet which texture cues related to object category and increase human-alignment on an adversarial texture vs shape categorization task (Geirhos et al., 2018), and four DinoV2 models, which are self-supervised ViT models also trained on ImageNet (Oquab et al., 2023).

We report how far a given model accuracy or trial-wise similarity to humans score is from the equivalent human accuracy or noise ceiling in units of standard deviations of the human accuracies (*STDs*_*toHumanAccuracy*_) or noise ceilings (*STDs*_*toHumanNoiseCeiling*_).

As expected, we observe a large gap in both accuracy and trial-level similarity between standard DNNs and human behavior (Figure 1c). Humans performed well on the task (*M* = 0.89, *STD* = 0.051, Figure 1c, x-axis). A noise ceiling was computed as the cosine similarity between a given participant’s trial-wise performance and the mean trial-wise accuracies across all other participants, computed in a leave-one-participant-out fashion and averaged across participants (*M* = 0.96, *STD* = 0.020, Figure 1c, y-axis). As with the human noise-ceiling, similarity to human behavior for each DNN model was computed as the cosine similarity between the vector of model responses across trials and the average human accuracy for each trial. We see much lower performance for ImageNet CNNs, with gaps in both accuracy (*M* = 0.70, *STDs*_*toHumanAccuracy*_ = −3.75) and trial-level similarity to humans (*M* = 0.85, *STDs*_*toHumanNoiseCeiling*_ = −5.47). ImageNet ViTs showed a similar gap compared to humans as CNNs in both accuracy (*M* = 0.71, *STDs*_*toHumanAccuracy*_ = −3.58) and trial-level similarity to humans (*M* = 0.86, *STDs*_*toHumanNoiseCeiling*_ = −4.99). The three CNNs trained on Stylized-ImageNet, which is designed to induce a shape bias, performed slightly worse than standard ImageNet CNNs on accuracy (*M* = 0.68, *STDs*_*toHumanAccuracy*_ = −4.21) and trial-level similarity to humans (*M* = 0.84, *STDs*_*toHumanNoiseCeiling*_ = −6.11). Finally, while the four DinoV2 models had the best average performance of any baseline model type in both accuracy (*M* = 0.73, *STDs*_*toHumanAccuracy*_ = −3.17) and trial-wise similarity to humans (*M* = 0.88, *STDs*_*toHumanNoiseCeiling*_ = −4.15), they still fell well short of human performance and alignment. These findings highlight the 3D shape processing gap that has been reported in the literature between standard DNNs trained on large corpuses of natural images and humans that we aim to address with the following work.

### Model Zoo

In order to identify what types of architectures and learning objectives increase alignment with human 3D shape inferences, we construct a model zoo of 3D Light Field Networks, resnet50 CNNs, and autoencoders trained with either single-view or multi-view learning objectives.

#### 3D Light Field Networks.

Light fields are an idealized representation that encodes the color of rays of light passing through every possible position in a scene at every possible orientation. A 3D Light Field Network (3D-LFN) (Sitzmann et al., 2021) instantiates this idea in a neural network by mapping from coordinates defining a ray through a volume to the RGB value of that ray. The mapping is implemented as a multi-layer perceptron (MLP), and once trained the weights of the MLP define the 3D light field, and thus the 3D shape, for a given object.

**Figure 2.**
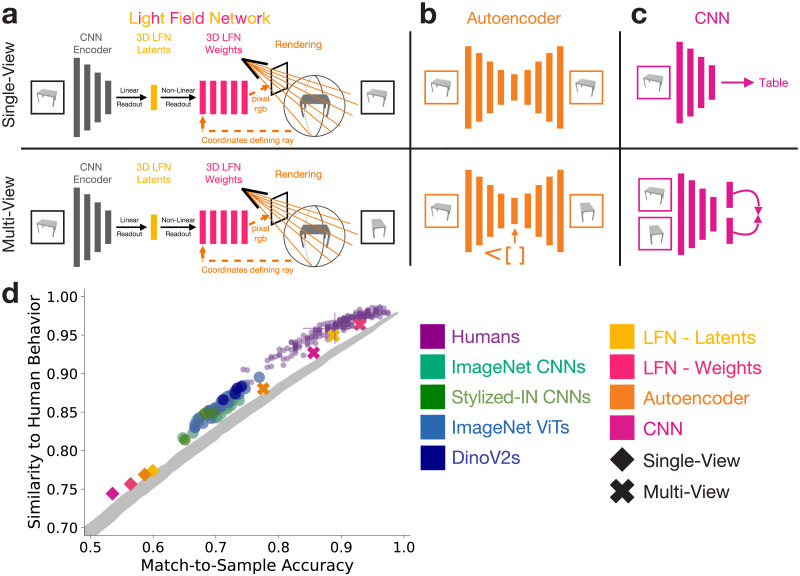
a.) Schematic for the 3D Light Field Networks. For all model variants, two versions were trained: a single-view model which operates over individual images and a multi-view version with a learning objective that encourages similar representations for two different views of the same object. b.) Schematic for autoencoder models. c.) Schematic for CNN models. d.) Multi-view DNN models are more aligned to human 3D shape judgements than the baseline DNNs. The 3D Light Field Networks achieve the highest accuracy and alignment to humans. Single-view versions of the same models trained on ShapeNet perform worse than the standard models, highlighting the important role of the multi-view learning objective in reaching human alignment. The shaded grey distribution is a random observer baseline that shows the expected range of trial-wise similarity to humans across accuracies if the correct and incorrect responses are randomly distributed across trials (see Methods - Random Observer Baseline).

The flow of information through 3D-LFNs (Figure 1a), during both training and inference, follows three steps (Figure 2a): 1.) Infer a set of shape latents (256D) from an RGB image, 2.) Map from shape latents to the neural field weights, 3.) Given a camera position, query rays from the neural field to render out an image. To infer shape latents from images (1), an RGB image is encoded using a CNN (resnet50) and 256D shape latents are linearly read out from the last convolutional layer. To map from latents to the neural field weights (2), a hypernetwork (a neural network trained to output the weights of another neural network) implemented using a series of two-layer MLPs with ReLU activation functions. The neural field itself is implemented as an eight-layer MLP with ReLU activation functions. The inputs to the field are coordinates defining a ray that passes through the space encoded by the field, and the output is the RGB value of the queried ray. To render the output image (3), a camera position and image plane are defined, and rays that pass from the camera through the image plane are sampled. The coordinates defining each ray are passed into the neural field, and the returned RGB value becomes the RGB value in the rendered image at the point where that ray intersects the image plane.

During training, a standard 3D-LFN is optimized end-to-end using a 3D multi-view learning objective. The input image depicts an object from one viewpoint, and the model’s objective is to render the same object from a different viewpoint defined by a camera matrix. The rendered image is compared to the ground-truth image of the object from the new viewpoint, and mean-squared-error between the rendered and ground-truth images provides the loss used to update network’s weights. The loss is back = propagated through the weights of the hypernetwork and CNN encoder. The CNN encoder is not pretrained, and its weights are entirely learned in tandem with the 3D-NF. During inference for test images, the latents and weights defining the 3D-LFN MLP are computed via a single feedforward pass through the model. Camera matrices can then be provided to render out the new object from the specified viewpoints, but the 3D-LFN latents and weights feature-spaces are computed without needing camera matrices.

This standard 3D-LFN training procedure is referred to as the multi-view learning objective using our nomenclature for these experiments. To create a single-view version of the 3D-LFN, where the architecture is identical but the model is not exposed to multiple viewpoints of the same object during training, the rendering objective for the model is made to be the same viewpoint as the input image. We use the shape latents and neural field weights as feature spaces for the subsequent 3D shape tasks.

#### Autoencoders.

Autoencoders are autoregressive DNN encoder-decoder architectures that use a series of encoder layers to map the input to a low-dimensional latent space, then use a series of decoder layers to reconstruct the input from the latent space (Figure 1b). We use a convolutional autoencoder architecture that encodes images to a 256D latent space using a a resnet50 encoder, then reconstructs the input image from the latents using a series of deconvolutional layers. This standard autoregressive autoencoder is the single-view learning objective variant.

To make a multi-view version of the autoencoder, we use the same architecture with two modifications. First, we adjust the model’s rendered output objective to be the input object from a different viewpoint instead of the input viewpoint. Second, we append an embedding of the output viewpoint camera matrix to the 256D latent space before the latents are passed into the decoder. This camera matrix embedding tells the model what the output viewpoint should be (similar to how 3D-LFNs use a provided camera matrix to define the output viewpoint).

For both variants, we use the 256D latent space as the feature space for the 3D shape tasks.

#### CNNs.

For our CNN models, we use a resnet50 architecture (Figure 2c). In the single-view case, we train the CNN to do 13-way ShapeNet category classification. We use the final convolutional layer (penultimate layer before classification layer) as the feature space for the 3D shape tasks.

For the multi-view case, we train self-supervised CNN using a resnet50 architecture and a modified MOCO contrastive loss (He et al., 2020) to learn similar embeddings for images depicting different viewpoints of the same objects (similar to Aubret et al., 2022). In standard MOCO CNNs, different image manipulations (e.g., translation, left-right flip, negative colors) are applied, and the model’s loss makes the embeddings computed from two modified versions of the same image as similar as possible relative to the embeddings computed for other images. We use this same scheme, but rather than applying image manipulations we provide two different viewpoints of the same ShapeNet object as input, compute the two embeddings in a siamese fashion with the same resnet50 encoder, then compute the loss over the embeddings for the two images. The model has a multi-view learning objective in that it must associate different viewpoints of the same objects as similar in its learned embedding space, but differs from the multi-view 3D-LFN and autoencoder in that the multi-view objective is not actually rendering out the input from novel viewpoints. We use the output embeddings as the feature space for the 3D shape tasks.

### Multi-View DNNs and Humans Make Similar 3D Shape Judgements

We trained all models outlined in the previous section on the ShapeNet dataset used for the match-to-sample task administered to humans, CNNs, and ViTs from the previous section. This training data includes 30565 objects from 13 object categories with 50 viewpoints per object, which are distinct from the test set of 8736 objects from which the stimuli for the match-to-sample experiments were rendered. The categories in the training and test sets are the same, but the specific objects are distinct. The shape latents and weights of the 3D-LFNs, the latent space of the autoencoders, and final pre-readout layer of the CNNs are extracted as separate sets of features, and the task is performed on all models using the same similarity-based measure applied to CNNs and ViTs.

For the multi-view 3D-LFN latents (Figure 2b), we see a marked improvement in both accuracy (*M* = 0.89, *STDs*_*toHumanAccuracy*_ = −0.040) and trial-wise similarity to humans (*M* = 0.95, *STDs*_*toHumanNoiseCeiling*_ = −.49). The multi-view 3D-LFN weights do even better (Figure 2b), reaching the mean human accuracy (*M* = 0.93, *STDs*_*toHumanAccuracy*_ = 0.78) and within one standard deviation of the human noise ceiling (*M* = 0.96, *STDs*_*toHumanNoiseCeiling*_ = 0.24). The multi-view CNN performance was within one standard deviation of human accuracy (*M* = 0.86, *STDs*_*toHumanAccuracy*_ = −0.67) and much closer to reaching the trial-wise similarity noise ceiling than any baseline model (*M* = 0.93, *STDs*_*toHumanNoiseCeiling*_ = −1.60). The multi-view autoencoder performed worse than all other multi-view models for both accuracy (*M* = 0.78, *STDs*_*toHumanAccuracy*_ = −2.25) and trial-wise similarity (*M* = 0.88, *STDs*_*toHumanNoiseCeiling*_ = −3.94), with performance and alignment to humans similar to the top baseline models.

The single-view 3D-LFN, autoencoder, and CNN all were the worst performing models, with much lower accuracies (*M* < 0.60, *STDs*_*toHumanAccuracy*_ < −5.73) and trial-wise similarity to humans (*M* < 0.77, *STDs*_*toHumanNoiseCeiling*_ < −9.28) than any of the baseline models.

Overall, these results show that the multi-view 3D-LFN supports 3D shape inferences that are aligned to humans for within-category comparisons, with both the latents and weights of the 3D-LFN falling within one standard deviation of both human accuracy and the human noise ceiling. Other multi-view models performed well, with the multi-view CNN approaching human-alignment and the multi-view autoencoder outperforming almost all of the baseline models. The extremely poor performance of the single-view variants of the same model highlights that a multi-view learning objective that encourages a viewpoint-invariant representation is a necessary condition for models to make similar 3D shape judgements as humans.

### 3D-LFNs and Humans Make Similar 3D Shape Judgements for “Adversarial” CNN-Defined Matching Judgements

Next, we used an “adversarial” stimulus-selection procedure to accentuate the failure modes of the baseline DNNs (i.e., where their performance deviates most sharply from humans). Using the 25 ImageNet CNNs, we filtered hundreds of thousands of potential within-category object pairs from ShapeNet to construct match-to-sample trials. We bin the object pairs according to the average CNN accuracy and sample from these bins to create a series of 5 adversarially-defined difficulty conditions. In the most difficult condition, the CNN average accuracy is 0.14 (*STDs*_*toHumanAccuracy*_ = −6.95) and the trial-wise similarity to humans is 0.38 (*STDs*_*toHumanNoiseCeiling*_ = −12.68). In the easiest condition, the CNN average accuracy is 0.68 (*STDs*_*toHumanAccuracy*_ = −3.95) and the trial-wise similarity to humans is 0.83 (*STDs*_*toHumanNoiseCeiling*_ = −5.81).

**Figure 3.**
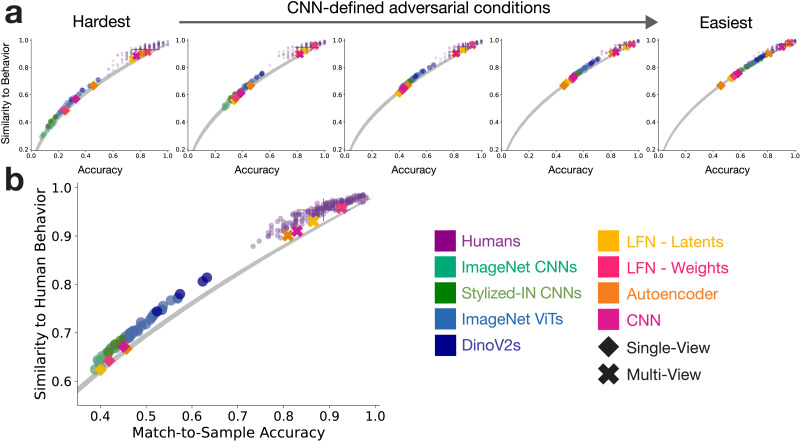
a.) In order to focus on the gap between humans and standard CNNs, many potential match-to-sample trials were filtered with the 25 ImageNet CNNs to select trials that fall in five difficulty conditions that adversarially accentuate the different between ImageNet-trained models and humans. The multi-view DNN features fall within the distribution of human response for all adversarially-defined conditions, including those where the baseline models perform much worse than humans. The shaded grey distribution is a random observer baseline that shows the expected range of trial-wise similarity to humans across accuracies if the correct and incorrect responses are randomly distributed across trials (see Methods - Random Observer Baseline). b.) Human and model performance and trial-wise similarity averaged across conditions.

Humans (*N* = 200) were largely unaffected by the CNN-defined difficulty conditions, showing high performance across all conditions (*M* > 0.83, Figure 3a). Out of the baseline models, ImageNet DinoV2s (*STDs*_*toHumanNoiseCeiling*_ > −6.90) performed best, followed by ImageNet ViTs (*STDs*_*toHumanNoiseCeiling*_ > −9.37), StylizedImageNet CNNs (*STDs*_*toHumanNoiseCeiling*_ > −12.10), and ImageNetCNNs (*STDs*_*toHumanNoiseCeiling*_ > −12.68), but all baseline models were far from human alignment (Figure 3).

Critically, 3D-LFNs were well-aligned with human behavior across difficulty levels for both latents (*STDs*_*toHumanNoiseCeiling*_ > −1.58) and weights (*STDs*_*toHumanNoiseCeiling*_ > −0.45). Across all difficulty conditions, the 3D-LFN weights were within 1 STD of the mean accuracy and noise ceiling compared to humans. The multi-view CNN (*STDs*_*toHumanNoiseCeiling*_ > −2.022) and autoencoder (*STDs*_*toHumanNoiseCeiling*_ > −2.09) models were also markedly more aligned with humans across difficulties than any of the baseline models. These results show that even when selecting trials where standard ImageNet models struggle, 3D-LFNs and other multi-view DNNs support 3D shape inferences similar to humans.

### 3D-LFNs and Multi-View DNNs Are Not Human-Aligned for Novel Shape Categories Outside Their Training Set

Finally, we test the extent to which the 3D-LFN and multi-view DNN models generalize to novel shape categories not included in training. Human 3D vision is able to represent novel shapes we have never encountered in our visual experience. If we come upon an abstract sculpture in a park, we are able to perceive its shape even if it occupies a new part of our shape space that has never been activated. In these experiments, one possibility is that the 3D-LFN and multi-view DNNs learn a broad shape space that can generalize to novel categories of objects they have not seen before. The alternative is that the 3D-LFN and multi-view DNNs learn a space that can generalize to novel exemplars of categories included during training (as we have shown in all previous experiments) but fail for new categories of shapes that fall outside their visual experience.

**Figure 4.**
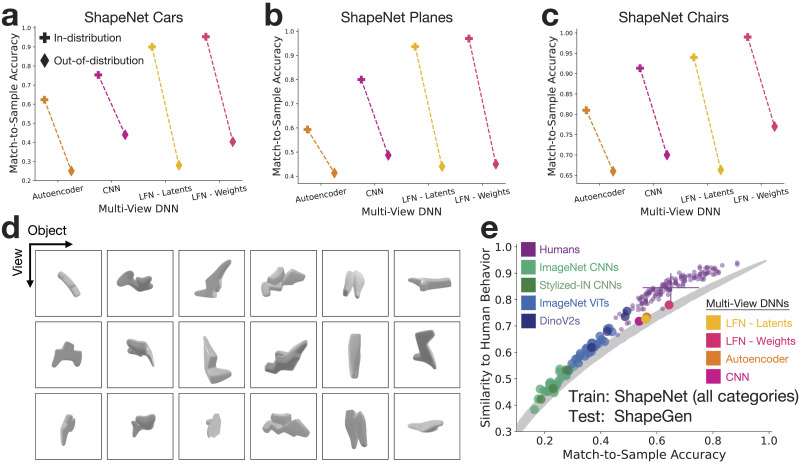
Out-of-distribution generalization for multi-view DNNs to novel object categories. For each ShapeNet category (a. cars, b. planes, c. chairs), all objects from that category were held out during training for new multi-view DNN models. Match-to-sample trials were adversarially selected for the held-out category using the CNN model zoo to accentuate the performance gap between baseline models and humans. We observe that the multi-view DNN models fail to generalize out-of-distribution to novel ShapeNet categories not included in their training distribution relative to the in-distribution multi-view models trained on all ShapeNet categories. d.) Example procedurally-generated ShapeGen stimuli. e.) Results testing the baseline model zoo and multi-view DNNs trained on all ShapeNet categories on the out-of-distribution ShapeGen stimuli. The shaded grey distribution is a random observer baseline that shows the expected range of trial-wise similarity to humans across accuracies if the correct and incorrect responses are randomly distributed across trials (see Methods - Random Observer Baseline). We observe that the ShapeNet-trained multi-view models reach human-level accuracy for ShapeGen stimuli, but the trial-wise similarity between humans and models is no higher than expected from randomly distributed correct trials.

To test the generality of the shape spaces learned by the 3D-LFN, multi-view autoencoder, and multi-view CNN, we first re-trained new versions of each multi-view model on ShapeNet holding out all cars, planes, and chairs in turn. We then constructed 3D match-to-sample experiments from only objects in each held-out category using the same adversarial stimulus selection procedure as earlier. With the CNN model zoo, we filter many possible object pairs for each held-out category and select the 150 pairs that best exemplify the performance gap between humans and standard DNN models. To evaluate generalization performance, we compare accuracies for the hold-one-category-out multi-view models described above to the multi-view models trained on all ShapeNet categories. For all new tasks, we find that all multi-view DNNs perform markedly worse when generalizing out-of-distribution to a new object category not included in the training set (Figure 4a–c).

The above results do not allow an equivalent comparison between models and humans given humans’ extensive prior experience with chairs, cars, and planes. To directly compare human and multi-view model generalization performance, we procedurally-generated a set of abstract 3D objects that should be similarly novel to both models and humans. We generated these objects using the ShapeGenerator (ShapeGen) extension for Blender. ShapeGen starts with a base shape, randomly extrudes mesh faces according to a few parameters, then applies a Catmull-Clark modifier to produce a smooth final object (Figure 4d). We used the same rendering procedure applied to ShapeNet to render images from these objects with viewpoints sampled from a sphere around the object, created 300 match-to-sample trials using the same CNN-based selection scheme applied to the held-out ShapeNet categories, and collected behavioral responses online (*N* = 140).

Human participants performed worse (*M* = 0.65, *STD* = 0.98) on this task than the ShapeNet tasks, but with a spread of performance from chance up to 0.88 (Figure 4d). This drop in human performance was expected as these shapes are complex, have protrusions that can be self-occluded from certain viewpoints, and humans have limited priors over the shape space to infer the unseen surfaces from a given view. Next, we evaluated whether the multi-view DNNs from previous sections trained on the full set of 13 ShapeNet categories generalize to make human-like 3D shape judgements on these abstract shapes. While the best performing multi-view model (3D-LFN weights) was very close to average human accuracy (*M* = 0.64, *STDs*_*toHumanAccuracy*_ = −0.066), all models fell below the human noise ceiling for trial-wise similarity (*STDs*_*toHumanNoiseCeiling*_ < −1.221) indicating that they did not make human-like judgements at a trial-wise level. This point is reinforced by all multi-view models falling within the random observer baseline (Figure 4d, grey distribution, see Methods - Random Observer Baseline) indicating that their trial-wise similarity to human behavior is no higher than expected if the responses were randomly distributed across trials.

Overall, these results show limitations in the out-of-distribution generalization performance of the evaluated 3D-LFNs and multi-view DNNs. While the multi-view models did achieve human-level accuracy for matching abstract ShapeGen objects, their trial-wise performance was not correlated with human trial-wise performance beyond what is expected by randomly distributed correct trials and the multi-view models show a marked drop in accuracy for ShapeNet categories held-out of the training set. It is important to note that the 3D object shape datasets we used to train our multi-view models are much smaller than what we expect human observers to experience during development, both in the number of unique object categories and the number of unique objects per category. With larger scale 3D shape training datasets, the difference between human observers and multi-view DNNs for abstract ShapeGen shapes may shrink or vanish.

## DISCUSSION

Our work has characterized the modeling choices that enable DNNs to approximate human-level 3D visual inferences, as well as the conditions under which this correspondence fails. We first replicate previous findings that standard (i.e., trained on ImageNet) DNN models fail to reach human performance on a within category match-to-sample task. We then construct a series of models consisting of 3D-LFN, autoencoder, and resnet50 models trained with both single-view and multi-view learning objectives. We find that only models trained with a multi-view learning objective come close to human performance, when evaluating on left-out objects from the categories these models were trained on. We then construct a series of ‘adversarial’ trials, designed to reveal the failures of standard DNNs. We find that multi-view models consistently fall within the human distribution for both accuracy and trial-wise similarity, vastly outperforming all other models. However, all multi-view models’ performance falls when generalizing to novel ShapeNet categories not included in the training distribution, and despite achieving human-level accuracy for abstract shapes the multi-view models trial-wise performance was not meaningfully correlated to human performance. These findings outline a tractable framework for studying human 3D vision within a deep learning framework, while highlighting limitations of current modeling approaches.

We emphasize that while a multi-view learning objective seems essential for models to achieve human-level 3D inferences under any conditions, our work points toward open questions about the additional contributions of model architecture and training objective. While all architectures trained with a multi-view objective approximated human performance, different models used different multi-view objectives. 3D-LFNs were trained to reconstruct objects from novel viewpoints (i.e., given input image from viewpoint A, render that same object from viewpoint B), while convolutional architectures were trained with a contrastive loss (i.e., represent an object from different viewpoints more similar than it is to different objects). As such, our observation that the 3D-LFNs best matched human accuracies and trial-wise patterns of behavior is ambiguous, because these 3D-LFNs differed from other multi-view models in both their architecture and their training objective. Future work is needed to adjudicate the role of architectures and learning objective in achieving human-like 3D shape judgements.

The importance of seeing objects from multiple viewpoints to support 3D visual learning, observed in our modeling results, is in line with research characterizing the visual inputs to developing human children (Smith et al., 2018). Frontward views of faces are the most common visual input in infant’s first 3 months, a pattern that continues but decreases over the following 9 months as seeing body parts of other humans and hands manipulating objects, especially during mealtimes, become increasingly common (Clerkin et al., 2017; Fausey et al., 2016; Jayaraman et al., 2015, 2017). Crucially, the viewpoints at which human toddlers see objects via their own self manipulation does not appear to be random, but curated to support visual statistical learning. Even for 4–7 month old infants, the ability to recognize the 3D shape of objects is most strongly linked to visual-motor manipulation abilities, which allow infants to expose themselves to many views of a novel object (Soska et al., 2010). Considering this empirical evidence, it is perhaps not surprising that computational models trained on standard datasets (e.g., Imagenet) do not spontaneously learn to represent objects in 3D, given that there is little in the training procedures to encourage a common representation across multiple viewpoints of the same object. Similarly, when we train models using such multi-view exposure to objects, they achieve human-level performance on within-category 3D shape judgements.

Recent work on learning online invariances in CNNs also supports the importance of multi-view exposure during training to learn 3D representations. Biscione and Bowers (2022) showed that CNNs can develop online invariances to common transformations, but only when the model’s training set included variation along the relevant dimension (Biscione & Bowers, 2022). They trained CNNs for object classification or same-different judgements on images rendered from ShapeNet with variation in the viewpoint, rotation, scale, translation, and brightness of the objects. Evaluating on a similar multi-view match-to-sample task as the one used here, they find that CNNs trained on images with a particular transformation learn invariance to that transformation. Most similar to the current work, they find that CNNs can learn to be viewpoint-invariant, but only when exposed to multiple viewpoints and rotations of the same object during training. While they do not evaluate the alignment between their models and humans, they suggest that special architectures may not be necessary to learn human-like invariances for viewpoint, position, and lighting provided the right training diet and task. These predictions are consistent with our findings that a variety of DNN architectures are aligned to human 3D shape judgements, but only when the learning objective enforces learning a common representation across two viewpoints of the same object. While a special architecture directly representing 3D space was most aligned to human behavior (3D-LFNs), a standard CNN architecture with a contrastive loss enforcing common representation across viewpoints showed a marked jump in alignment to human 3D shape judgements. Future work will further adjudicate the role of architectures, special or otherwise, in achieving human-like 3D shape judgements, but exposure to multiple-viewpoints of objects during training is evidently essential.

This work shows how much of the performance gap can be closed between DNN and human abilities at matching objects across view points. However, this does not mean that these models necessarily implement the same processes as humans. As discussed next, a gap still exists in generalization beyond the training set, and the models remain biologically implausible.

Even the best performing models struggled to generalize to novel shapes not included in their training distribution. DNNs’ failure to generalize beyond their training set has been well characterized in the computer science literature (Geirhos et al., 2020; Recht et al., 2019; Zhang et al., 2021) and these generalization failures stem from DNNs’ tendency to overfit on their training set and interpolate within the training distribution at test time. This limitation is the driving force in the ongoing quest for larger and larger datasets to train DNNs so that more information is within-training-distribution. Through this lens, the mixed generalization performance of the multi-view DNNs is broadly consistent with expected DNN behavior. It is important to note that even the full ShapeNet dataset we use for training here is much smaller, both in number of object categories and number of unique objects per category, than what humans are exposed to during development. For this reason, while we observed limitations in models’ out-of-distribution generalization for this set of experiments, it’s possible the model generalization will improve with much larger 3D shape training datasets. It will be important for future work to test generalization to novel shape categories with DNNs trained on much larger datasets, and it remains possible that modeling frameworks other than DNNs may be necessary in the long run to achieve human-level generalization.

Many aspects of the models we have evaluated here are biologically implausible. For example, 3D-LFNs are models that regress 3D shape latents from a CNN, map the latents to the 3D field space, then use a series of graphics techniques to render an image of the represented object from a viewpoint specified by a provided ground-truth camera matrix. There is no reason to expect that these graphical techniques—such as casting rays through an image-plane to render an pixel-level RGB values of the depicted object—are implemented by biological neurons. Additionally, these methods require ground-truth information about camera position—a form of direct 3D supervision which is never provided to human observers. We are not proposing that the brain is using computer graphics techniques such as rendering and casting rays (or backpropogation for that matter) to learn 3D representations; this is simply one avenue to enforce 3D representations in models to produce human-aligned 3D shape judgements. An important direction for future research is to identify novel learning objectives that support 3D learning, perhaps via analysis-by-synthesis techniques, that can be implemented by neurons without standard graphics tools.

The 3D shape representations in the multi-view DNNs described in this work are implicit 3D representations encoded in the patterns of artificial neurons in DNNs. Given the biological implausibility of several components in our models, we do not make a claim here that humans are using a 3D shape representation of a similar format to the ones in our models to complete the 3D matching tasks. In addition to implicit representations encoded in neurons, other possibilities for the format of the human 3D shape representation include metric 3D representations such as depth or surface normal maps that encode local aspects of geometry across a scene or object, Biederman’s recognition-by-components model (Biederman, 1987) which uses local image features to perform 3D shape tasks, optic flow maps that track how objects move over time (Stewart et al., 2022, 2024), correspondence-based measures (e.g., 3D point matching algorithms in computer vision; El Banani et al., 2024), not to mention explicit 3D shape representations such as triangular meshes and point clouds. An important direction for future work is determining the format of representation used by humans to complete 3D matching tasks and encapsulating such findings in computational models.

What kind of constraints should be used to design more biologically plausible models of 3D perception? The field has long considered late stages of processing within the ventral visual stream (VVS) as representing object shape (DiCarlo et al., 2012). More recent work suggests that the VVS may, in fact, simply provide a basis space of texture-like representations which can be used to infer shape-level object properties (Jagadeesh & Gardner, 2022). This claim inverts a common critique of standard DNNs (e.g., Geirhos et al., 2018), suggesting that the VVS, like CNNs, is ‘texture-biased.’ As such, it’s possible that while some 3D shape properties might be represented within the VVS, there are neuroanatomical structures beyond the VVS which are critical for this ability. The dorsal stream, for example, has been implicated in 3D shape processing (Sereno et al., 2002; Van Dromme et al., 2016), suggesting that the sorts of structured 3D representations captured by DNNs may share capture more variance in dorsal neural responses than standard DNNs. In recent work, Bonnen et al. (2021) has shown that medial temporal cortex (MTC), downstream from the VVS, plays a causal role in 3D object perception (Bonnen et al., 2021). Moreover, these inferences depend on reliable visuospatial sampling policies (Bonnen et al., 2023), suggesting that there are behavioral signatures of shape inferences. These data provide clear neuroanatomical and algorithmic constraints on models which aim for more human-like 3D object perception.

In summary, we have shown that DNNs with multi-view learning objectives can approximate human 3D shape inferences for object types included in the DNN training distribution, but show mixed results when generalizing to out-of-distribution shapes. The limitations of standard DNN models are well recognized, yet it has been unclear what model components (e.g., architecture, training objective, or dataset) leads to their misalignment with human 3D shape inferences. The field has historically focused on the role of model architecture (Richards et al., 2019; Yamins & DiCarlo, 2016), yet in recent years the role of training data has become increasingly prominent (Conwell et al., 2022). Our work extends this line of reasoning into the domain of 3D shape perception by independently manipulating the effects of architecture and dataset. Of course, there are obvious differences between the current 3D models and biological brains, but resolving these engineering challenges is a critical step in demonstrating that DNNs are capable of human-aligned 3D inference. We leave it to future work to use neural and behavioral constraints, DNN approaches, and other modeling frameworks, such as probabilistic programming, to design and evaluate more biologically plausible computational models of human 3D object perception.

## METHODS

### 3D Shape Datasets

ShapeNet is a large collection of 3D object meshes drawn from a variety of manmade categories. We use the 13 largest ShapeNet categories, with a total of 39301 objects across all categories. The categories are: airplanes, tables, cars, lamps, chairs, stereos, benches, phones, guns, couches, cabinets, boats, monitors. 30565 objects were assigned to a training set for learning 3D models, and 8736 were assigned to the test set. All objects used in the match-to-sample tasks for humans and models were drawn from the test set for all experiments. Using Blender (Blender Online Community, 2018), we rendered 50 images of each object from viewpoints randomly sampled from a sphere around the object. The objects were rendered matte grey without color or textures to emphasize shape processing in the models and experiments.

ShapeGenerator (ShapeGen) is a tool for procedurally generating abstract objects in Blender (https://blendermarket.com/products/shape-generator). It provides a simple interface through which a user can generate abstract objects by fusing together several simpler shapes of different sizes, each of which can be rotated, beveled, smoothed, or extruded according to predefined user specifications. To generate the shapes in our dataset, we predefined ranges for several parameters and sampled values uniformly within those ranges, which provided smooth and interesting variation across shapes. More concretely, our shapes were generated by taking a single cube as a base object (whose initial length and width is set by a random seed), extruding a random face on that object between 5–10 times (each time at a random angle, and random length), and rounding the edges by applying a catmull-clark modifier to the resulting mesh, to create a smooth shape. 10000 objects were generated. As with ShapeNet, all objects used in the match-to-sample tasks were drawn from the test set, Blender was used to render 25 viewpoints of each object, and objects were rendered matte grey. The viewpoints for ShapeGen objects were sampled randomly from a sphere around each object.

### Human Behavioral Experiments

Human behavioral experiments were conducted online using Prolific. Participants were each paid $15/hr for participating. Experiments were approved by the MIT Committee on the Use of Humans as Experimental Subjects. Participants were notified of their rights before the experiment, and were free to terminate participation at any time by closing the browser window. Experiments consisted of an initial set of instructions, 6 practice trials with feedback, and 150 main trials with no feedback. Participants were not able to progress to the main experiment until they successfully completed all 6 practice trials. Each experiment took an average of 15 minutes for participants to complete. Experiments were presented to participants using a standard JsPsych toolkit (de Leeuw et al., 2023). Participants were screened for participation in prior studies in this paper, so each participant only appears once and in just one experiment.

For all experiments, trials were constructed by assigning two objects to a pair. For each pair of objects, two trials were constructed, one with object A as the sample and target and B as the lure, and one vice versa. In the behavioral experiments, participants were split into two batches and each batch only saw one trial for a given object pair ensuring that all objects were novel for each trial.

### Model Zoo of Standard DNNs

The model zoo of standard DNNs consisted of 25 pretrained convolutional neural networks (CNNs) and 25 pretrained visual transformers (ViTs).

The 25 CNNs were pretrained for object classification on ImageNet and downloaded from PyTorch (pytorch.org). The models used were: alexnet, densenet121, densenet161, densenet169, densenet201, resnet101, resnet101wide, resnet152, resnet18, resnet34, resnet50, resnet50wide, resnext101, resnext50, shufflenetv2, squeezenet1.0, squeezenet1.1, vgg11, vgg11bn, vgg13, vgg13bn, vgg16, vgg16bn, vgg19, vgg19bn. For all analyses, the unit activity for the penultimate layer before the classification layer were extracted.

The 25 ViTs were pretrained on ImageNet and downloaded from the timm (timm.fast.ai) package. As with the CNNs, all models were pretrained for object classification on ImageNet. The models used were: convit_base, convit_small, convit_tiny, mvitv2_base, mvitv2_base_cls, mvitv2_huge_cls, mvitv2_large, mvitv2_large_cls, mvitv2_small, vit_base_patch16_224, vit_base_patch16_clip_224, vit_base_patch32_clip_224, vit_base_patch8_224, vit_base_r50_s16_224, vit_large_patch14_clip_224, vit_large_patch32_224, vit_relpos_base_patch16_224, vit_relpos_base_patch16_clsgap_224, vit_relpos_medium_patch16_224, vit_relpos_medium_patch16_rpn_224, vit_small_patch16_224, vit_small_r26_s32_224, vit_srelpos_medium_patch16_224, vit_srelpos_small_patch16_224, vit_tiny_r_s16_p8_224. For all analyses, the unit activity for the penultimate layer before the classification layer were extracted.

### 3D Light Field Networks

The essence of a neural field generally is a neural network model that takes some set of coordinates as input and outputs a property at those coordinates. Common examples of fields are magnetic or gravitational fields, both of which encode the continuous function of magnetic or gravitational force across space. In computer graphics, 3D shape fields are learned by mapping from coordinates in space (e.g., xyz, ray coordinates) to color or volume properties at that location. Once the neural network is trained, an image depicting the encoded space from a given viewpoint can be rendered by querying the neural field to recover shape/color. The most commonly used neural fields, neural radiance fields (NeRF) (Mildenhall et al., 2021), take xyz coordinates as input and output RGB and volume density at the queried coordinate. The field is then queried using a volumetric renderer to produce images of the scene from a given viewpoint. NeRFS have been used in computer graphics to create photorealistic encodings of objects and scenes by overfitting the field with many (>100) views from an individual scene. More relevant for modeling perception, a class of neural fields called conditional neural fields compute the continuous 3D function for objects from images (conditional because the field is conditioned on the input image). The models used here fall into this latter camp.

3D Light Field Networks (3D-LFNs) (Sitzmann et al., 2021) are models that compute a continuous function defining the light field of a volume in space using neural networks. 3D-LFNs follow three stages, each with their own architecture: 1. Inferring 3D shape latents from images using a CNN encoder, 2. Predicting the weights of the neural light field from the shape latents using hypernetworks, and 3. Using the neural light field to render an image from a given camera viewpoint. These models are part of a broader class of models in computer vision called conditional neural fields (Xie et al., 2022), which estimate neural fields from images rather than fit them directly to a given scene with many viewpoints, as in common in computer graphics (Xie et al., 2022).

The inference stage (1) of the 3D-LFNs used here was implemented as a resnet50 CNN encoder. The final classification layers were removed, and a linear layer was used to map from the final convolutional layer to a 256D fully-connected layer that represents the 3D latent space for the model. To map from shape latents to the weights of the 3D light field (2), we used a hypernetwork. A hypernetwork is a class of neural networks used in metalearning (Ha et al., 2017) that is trained to predict the weights of another neural network. The hypernetwork is implemented as a series of two layer multi-layer perceptron (MLP) with 256 units per layer that takes the 256D shape latents as inputs and outputs the weights for one layer of the neural field. There is one hypernetwork for each adjacent pair of layers in the neural field. The neural light field itself is implemented as an 8 layer MLP. The first layer input is a six dimensions for the plücker coordinate defining a ray through the scene, the output layer is three dimensions for the RGB value of the ray, and the hidden layers have 256 hidden units. For the rendering procedure during training, a ground-truth camera matrix is provided, an image-plane defined at a fixed distance between the camera and object, and rays are queried such that they pass through the camera plane from the camera position. One ray is queried per pixel in the output render, and the RGB value returned by the neural field for a given ray becomes the RGB value for the pixel where that ray intersects the image plane. The loss for training the model is the mean-squared-error between the output render and the ground-truth image of the object from the same camera position. The loss is backpropogated to update the weights in the hypernetworks and CNN encoder.

For all 3D-LFN analyses, we extracted the shape latents and weights defining the 3D-LFN MLP as features. The 3D-LFN latents are a 256D fuly-connected layer linearlly read out from the CNN encoder. The 3D-LFN weights were dimensionality reduced to a 150D feature-space using PCA before subsequent analysis.

### Autoencoders

We implemented 2 different types of autoencoders. The first is a standard autoencoder that takes an image as an input, runs it through a resnet50 encoder and maps to an 256D latent space, then uses a series of deconvolutional layers to map from the latent space back to reconstruct the input image. In our experiments, we use this as a single-view variant of the autoencoder. We also train a multi-view autoencoder that identical to the single-view version, but modified to support a multi-view learning objective. In this version, the input image and output rendering target are two different viewpoints of the same object. The camera matrix embedding appended to the latent space defines the target output viewpoint the deconvolutional block should reconstruct. These models were implemented with custom PyTorch code.

For all autoencoder models, the match-to-sample tasks were completed using the same similarity-based measure as all other models applied to the ND latent space.

### Multi-View CNN

For the multi-view CNN, we used a standard resnet50 CNN architecture with a modified Momentum Contrast (MOCO) style learning objective (He et al., 2020). In standard MOCO models, a target image is augmented in two ways (possible manipulations: random cropping, random cropping + resizing, color jittering, random greyscale conversion, random horizontal flip, random gaussian blur). Each augmented image is passed through the same resnet50 and mapped to an 2046D output latent space. The model’s learning objective is to make the latents for the two ablated versions of the input image as similar as possible (positive samples) relative to augmented versions of other images in the same minibatch (negative samples). To modify this for a multi-view learning objective, we instead provide the model with two images of the same object from different viewpoints (without any augmentations). The model’s objective then becomes to make the two output latents for different viewpoints of the same object as similar as possible relative to negative samples from un-augmented images of other objects in the minibatch. We used a modified version of the implementation in the PyContrast repository (github.com/HobbitLong/PyContrast). To train a single-view version of the CNN, we train a ShapeNet classification resnet50 (13 classes) using a cross-entropy classification loss.

### Model Alignment to Human Behavior

Human and model behaivor was compared in a trial-level fashion using cosine similarity. Average trial-level human accuracies were computed across participants for each trial, producing a vector of accuracies. For models, the binary correct/incorrect performance for each trial was also arranged into a vector over trials. Similarity to Human Behavior was computed as one minus the cosine distance between the human and model accuracy vectors.

The human noise-ceiling was computed using a similar procedure within the human data. In a leave one out fashion, the similarity between the binary correct/incorrect vector for one participant and the average accuracies for the remaining participants was computed. This is done for each participant, and the average across participants is the noise-ceiling for the model comparisons.

### Random Observer Baseline

As human accuracy increases on our 3D matching tasks, there are less trials for discrepancies between the models and humans for the trial-wise similarity measure to detect. At a minimum, we want to ensure that each model’s trial-wise similarity to humans is higher than would be expected if the correct/incorrect responses were randomly distributed over trials. To provide such a reference baseline when plotting these results, we compute a random observer baseline distribution that shows the expected trial-wise similarity to humans across accuracies if the correct and incorrect responses were randomly distributed over trials. For each behavioral experiment, we simulated 1000 participant responses with their corresponding correct/incorrect responses randomly distributed over trials for each possible accuracy (0.01 to 1 at intervals of .01). We computed trial-wise cosine similarity between each of the 1000 simulated subjects/models to the average human behavioral accuracies. The shaded baseline distribution plotted in each results figure is the 95% confidence interval over the 1000 simulations for each accuracy.

## ACKNOWLEDGMENTS

We thank Kevin Smith and Ratan Murty for helpful feedback on this work.

## FUNDING INFORMATION

This work was supported by National Institutes of Health grant DP1HD091947 awarded to Nancy Kanwisher, Office of Naval Research MURI grant PO #BB01540322 awarded in part to Joshua B. Tenenbaum, and the Center for Brains, Minds, and Machines (CBMM) funded by NSF STC award CCF-1231216.

## AUTHOR CONTRIBUTIONS

TPO, JBT, and NK conceived of the research. TPO, NK, and TB designed the experiments with input from JBT. YF and TPO collected the human behavioral data. TPO constructed the models with assistance from AT and VS. TPO conducted the modeling and behavioral analyses with input from TB. TPO, NK, TB, and JBT interpreted the results. TPO and TB wrote the paper with input from NGK, JBT, YF, and VS.

## DATA AVAILABILITY STATEMENT

The software packages and datasets used to create all stimuli are available online:

- ShapeNet.v2 Dataset (shapenet.org)
- Blender (blender.org)
- ShapeGenerator (https://blendermarket.com/products/shape-generator)

The following Python packages were used for the modeling:

- All analyses were completed using Python (python.org)
- Code to train Light Field Networks is available on GitHub (github.com/vsitzmann/light-field-networks)
- The ImageNet CNNs are available in Pytorch (pytorch.org)
- The ImageNet ViTs are available in timm (timm.fast.ai)
- The Multi-View CNN was trained with a modified version of PyContrast (github.com/HobbitLong/PyContrast)
- The DinoV2s are available on GitHub (https://github.com/facebookresearch/dinov2)
- The Stylized ImageNet models are available on GitHub (https://github.com/rgeirhos/texture-vs-shape)

Behavioral data from 3D-LFNs and Humans Make Similar 3D Shape Judgements for “Adversarial” CNN-Defined Matching Judgements section and similar data for evaluating multi-view consistency between models and humans is available at https://huggingface.co/datasets/tzler/MOCHI (Bonnen et al., 2024).
